# A Practical Quality Assurance Procedure for Data Acquisitions in Preclinical Simultaneous PET/MR Systems

**DOI:** 10.1007/s11307-022-01787-1

**Published:** 2022-12-07

**Authors:** Alan Courteau, John McGrath, Paul Michael Walker, Benoît Presles, Ruslan Garipov, Alexandre Cochet, François Brunotte, Jean-Marc Vrigneaud

**Affiliations:** 1 ImViA Laboratory, EA 7535, University of Burgundy, 21000 Dijon, France; 2 Georges-François Leclerc Cancer Centre, Unicancer, 21000 Dijon, France; 3 MR Solutions Ltd, Guildford, GU4 7WA UK; 4 University Hospital Centre François Mitterrand, 21000 Dijon, France

**Keywords:** PET, MRI, Preclinical imaging, Quality control, Simultaneous imaging

## Abstract

The availability of preclinical simultaneous PET/MR imaging systems has been increasing in recent years. Therefore, this technique is progressively moving from the hands of pure physicists towards those of scientists more involved in pharmacology and biology. Unfortunately, these combined scanners can be prone to artefacts and deviation of their characteristics under the influence of external factors or mutual interference between subsystems. This may compromise the image quality as well as the quantitative aspects of PET and MR data. Hence, quality assurance is crucial to avoid loss of animals and experiments. A possible risk to the acceptance of quality control by preclinical teams is that the complexity and duration of this quality control are increased by the addition of MR and PET tests. To avoid this issue, we have selected over the past 5 years, simple tests that can be easily and quickly performed each day before starting an animal PET/MR acquisition. These tests can be performed by the person in charge of the experiment even if this person has a limited expertise in instrumentation and performance evaluation. In addition to these daily tests, other tests are suggested for an advanced system follow-up at a lower frequency. In the present paper, the proposed tests are sorted by periodicity from daily to annual. Besides, we have selected test materials that are available at moderate cost either commercially or through 3D printing.

## Introduction

The integration of positron emission tomography (PET) in magnetic resonance (MR) systems brings a wide range of innovative applications, not only in the clinical domain, but also for small animal explorations [1]. Indeed, when used as a morphological modality, magnetic resonance imaging (MRI) offers a better tissue discrimination than X-rays computed tomography (CT) without exposing the animal to ionising radiation, which is a strong asset for longitudinal studies. In addition, nuclear magnetic resonance (NMR) spectroscopy and functional MR imaging (fMRI) sequences allows investigators to study a large panel of physiological parameters ranging from brain metabolites concentration to tissue perfusion or oxygenation [2, 3]. On the other hand, PET is a sensitive molecular imaging technique capable of *in vivo* radioactivity quantification when used with adequate calibration processes [4]. Compared to sequential procedures, simultaneous PET/MR acquisitions lead to spatially and temporally consistent data, limiting registration error and physiological bias. In addition, the dry magnet technology has drastically improved the compactness of MR systems, allowing their installation in research facilities that were not designed to host cumbersome imaging devices [5, 6]. For these reasons, compact integrated PET/MR systems are going to play an increasingly important role in preclinical research.

The integration of PET in MR systems has been made possible since the late 2000s thanks to the emergence of avalanche photodiodes (APDs) [7] and silicon photomultipliers (SiPMs) [8]. During the last decade, several academic and industrial research teams have focused on the development of simultaneous PET/MR instruments for small animals [5, 9–14], increasing the number of commercially available fully integrated scanners.

Although continuous instrumentation improvements have been made, PET/MR systems remain complex and might suffer deviations of their performance under the influence of external factors or mutual interferences between subsystems [15]. Therefore, in order to (1) avoid any issues during *in vivo* procedures, (2) maintain the image quality, and (3) ensure the quantitative aspect of PET and MR data, the characteristics of both subsystems must be closely monitored. As no standardised procedures have been issued for this task, our goal is to present the practical basis of a routine quality assurance (QA) dedicated to integrated preclinical PET/MR systems. The principles behind the proposed routine procedures were to select tests that are easy to perform especially in the daily workflow of the facility.

Our procedures are divided in two sections. The *basic* section includes measurements that can be applied by anyone who knows the basics of the system (i.e. PET and MR acquisitions and reconstructions). These tests are likely to detect the most common problems. The *advanced* section requires a deeper understanding of the two modalities and advanced knowledge in image processing. The procedures have been developed on our site where an integrated 7-Tesla compact PET/MR has been operated for 5 years (MR Solutions Ltd., Guildford, UK). The characteristics and performances of our system were previously reported [5].

## Basic QA Program

### General Principles for the Basic QA

The basic QA program intends to check PET/MR hardware and software main components. Despite its apparent simplicity, it has proven to be effective in detecting the most common issues we have faced. The daily checks are performed before starting the *in vivo* experimentation, or at least once a week if the system has not been used. The results and user comments are stored in a spreadsheet.

The procedure requires simple test objects (phantoms) mimicking the presence of a small animal or designed to measure a specific system characteristic. Regarding software, an NMR spectra visualisation tool and a basic image processing software are needed, which are usually provided by the supplier. The proposed daily tests do not require the use of radioactivity. For the test requiring radioactive fluorine, ^18^F can be under any chemical form.

It is important to note that unlike most of the performance assessment tests found in the literature, all the proposed tests are performed with the PET and the coil installed inside the magnet bore.

### Basic Daily Tests

#### Magnet Checks

When arriving in the laboratory, the QA operator ensures that the MR hardware components are properly functioning. In the imaging room, the presence of the principal magnetic field (further referred to as B0) is attested safely using a simple compass. The procedure is continued with a visual inspection of the overall system including its most fragile and frequently handled accessories like the radiofrequency (RF) coils, cables, and connections. This inspection may reveal some minor material damages that need to be addressed.

#### Dry Magnets-Specific Checks

For dry magnets (i.e. in the absence of liquid helium in the magnet’s cryostat), the solenoid’s inner temperature should be verified, as well as gaseous helium pressure of the cryocooler system. Dry superconducting magnets partly maintain their inner temperature by the mechanical action of a device called a *cold head*. This cold head emits a pumping noise at a specific frequency, typically at 1 Hz. Any variation of this acoustic noise may indicate a chiller system issue.

#### Animal Care Materials

The working condition of the materials dedicated to the animal’s anaesthesia or physiological monitoring is tested. This includes bed heating devices, temperature probe, cardiorespiratory signal sensors and their related analogue-to-digital conversion and record systems, as well as the rather fragile optical fibre connections often used for their MR compatibility. These materials can be tested in the absence of an animal by connecting the required gating materials and launching a blank cardiorespiratory acquisition. A flat electrocardiogram trace should be displayed by the visualisation software. This simple check is usually sufficient to rule out a connection issue within the cardiorespiratory signal acquisition chain. Because animal care devices can interfere with image quality results, we recommend removing them from the FOV before performing the daily image acquisitions presented thereafter.

#### MR and PET Background Acquisitions Test

This simplified test aims at controlling the MR and PET response in the absence of any object or radioactive source in the field of view (FOV). In practice, the user proceeds to a live observation of the MR noise spectrum and PET counting rates on every detection head during a simultaneous acquisition.

**Fig. 1:**
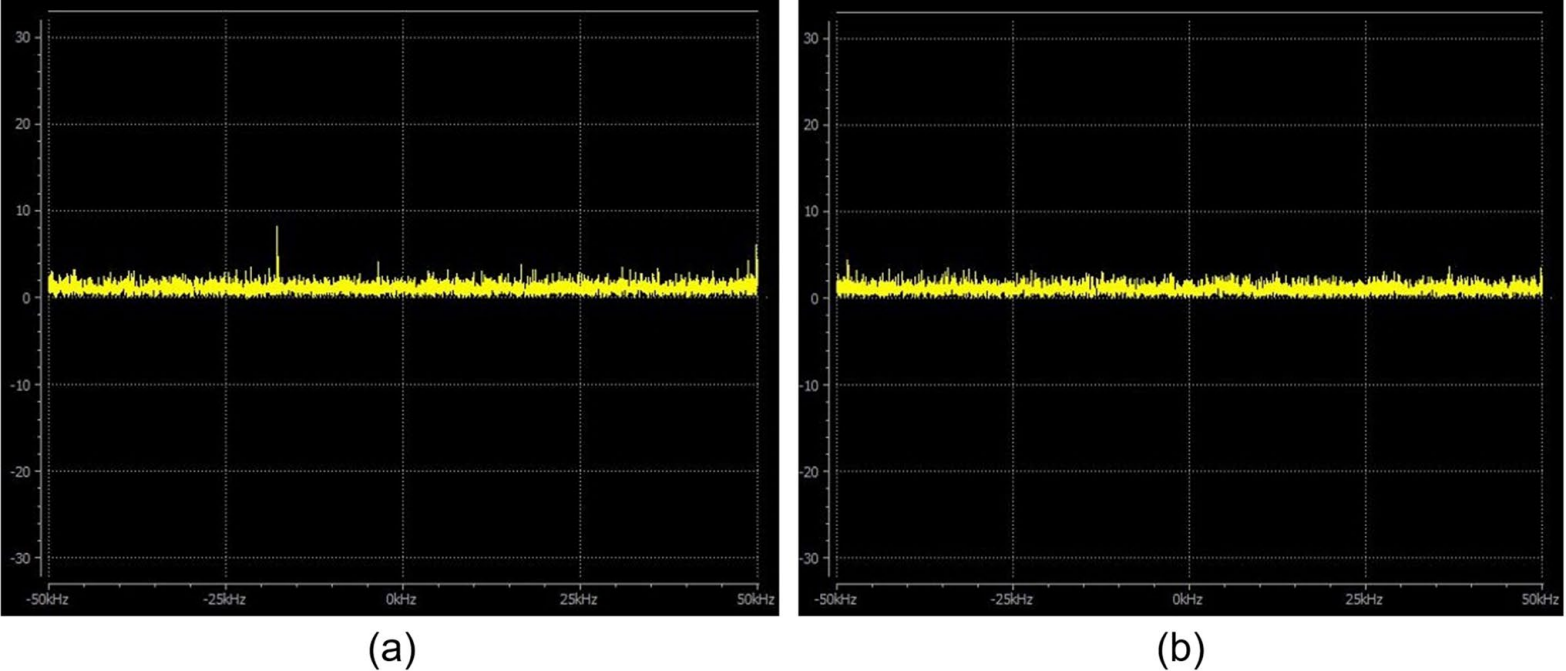
MR magnitude noise spectrum (kHz) obtained with a rat body quadrature coil in reception mode. **(a)** Unexpected peaks are visible, mainly at -18.5 kHz. This was caused by an unshielded high-definition multimedia interface wire in the imaging room. This unwanted peak will be materialised on the image in the form of white streaks artefacts particularly visible with gradient echo sequences. **(b)** Same acquisition after the cable was unplugged

The MR noise spectrum is acquired during 3 min using the receiver mode (i.e. no radiofrequency emission) of a quadrature coil. A broad bandwidth of ± 50 kHz around the central frequency is used. The spectra analysis does not require complex signal interpretation. The MR noise mean amplitude is reported and compared to the previous values. The operator systematically reports the unexpected peaks which show up within the white complex Gaussian noise expected in quadrature detection [16]. These peaks may indicate an interference between the RF coil and the surrounding electromagnetic environment, which can be a source of image artefacts. This is particularly important when the system environment has been modified (e.g. installation of new materials in the room) as presented in Fig. 1.

Simultaneously with the MR noise spectrum acquisition, the operator launches a 1-min PET background acquisition to ensure that the counting rates in the absence of any source is similar on every detector head. The background counting rates are mainly caused by the natural radioactivity of the LYSO scintillator. The count rates and the temperature of the SiPMs, assuming that the latter is available, are compared to a reference value, established during the acceptance procedure or supplied by the manufacturer.

#### MR Image Acquisition and Radiofrequency Coil Check Test

The purpose of this test is to verify the ability of the MRI system to complete gradient echo (GRE) and spin echo acquisitions without distortion or artefacts.

**Fig. 2:**
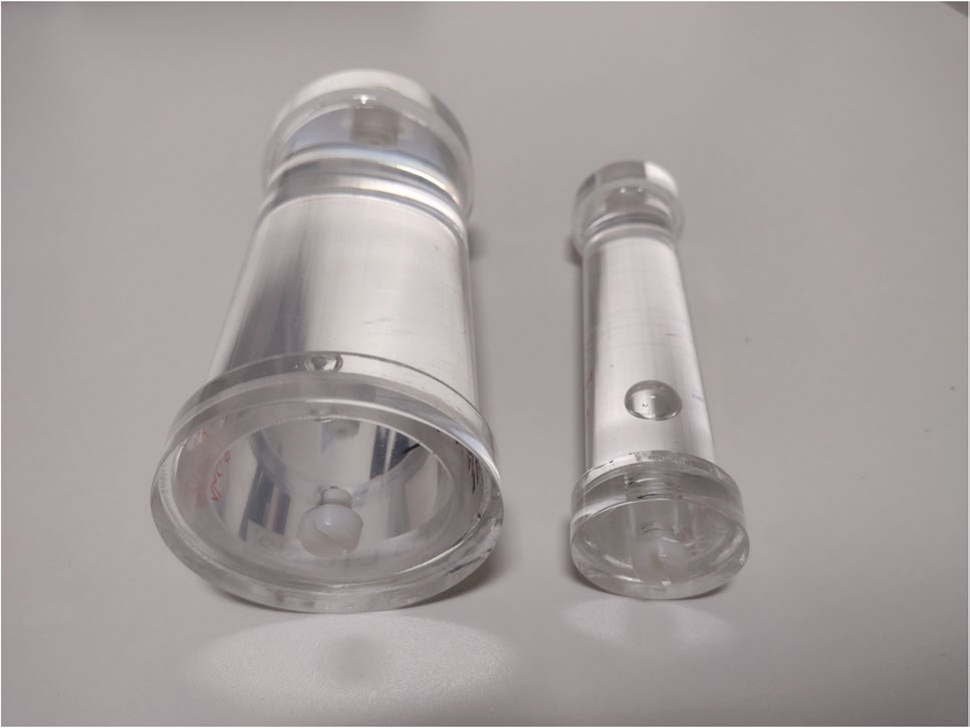
Picture of the mouse-sized cylinder phantom (on the right side) used for several PET and MR tests. This phantom is here presented near a rat-sized version (on the left side). The phantoms are 10-cm long to fit our PET axial FOV. The two phantoms have a diameter of 2 cm and 4 cm respectively for the mouse and rat

A simple water cylinder mimicking a mouse, like the one shown in the left side of Fig. 2, is installed in the appropriate bed and centred in the FOV. The automatic bed movement and bed emergency stop button are tested at this step.

**Fig. 3:**
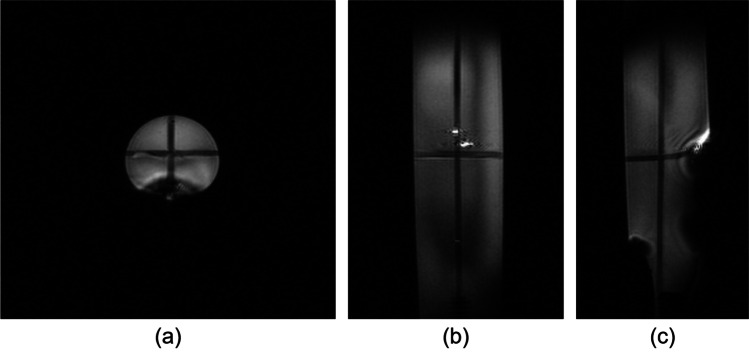
Example of multiplanar GRE scout views of the mouse-sized cylindrical phantom acquired in 15 s. **(a)**, **(b)**, **(c)** The transaxial, coronal, and sagittal planes. The visible deformation is caused by a non-MR compatible 27-G catheter voluntary introduced in the FOV where the caudal vein would have been located during an *in vivo* acquisition

Once the phantom is installed and centred, the operator launches three pre-scan MR calibrations (central frequency, RF emission, active shimming) prior to the acquisition of a multiplanar scout view. This GRE sequence is fast, about 15 s, and is efficient to detect many MR issues, as illustrated in Fig. 3 in the case of a B0 distortion.

Then, the American College of Radiology (ACR) single-image signal-to-noise ratio (SNR) method is applied [17]. A T1-weighted (T1w) fast spin echo (FSE) axial image is acquired on the mouse-sized phantom filled with water with the following acquisition parameters: repetition time (TR) = 1000 ms, echo time (TE) = 20 ms, image matrix = 256 × 256 pixels, three signal averages, and FOV = 60 mm × 60 mm × 2 mm to get an image size of at least twice the phantom diameter. To prevent SNR-temperature dependency effect, the room and the MR gradients coil temperatures should be monitored, and the acquisitions are carried out with no bed heating.

**Fig. 4:**
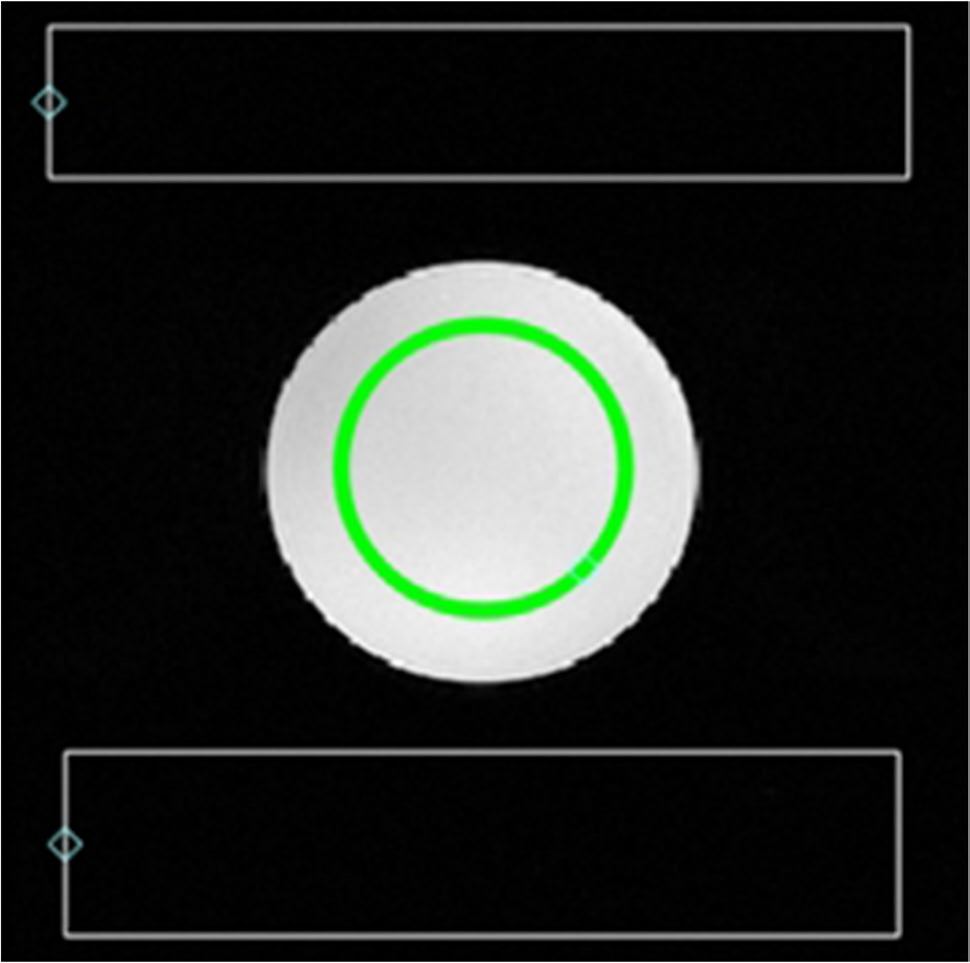
Illustration of the SNR measurements. The green circular ROI is dedicated to the signal measurement, whereas the white rectangular ROIs, drawn outside the ghosting region, are used to measure the noise. Any artefact is reported

The image analysis is performed as follows. First, the presence of artefacts is investigated while varying the image window. A slight ghosting effect is normal in the phase-encoding direction, but any other artefact or image distortion should be addressed. Two rectangular air regions of interest (ROI) are drawn outside the ghosting region (noise regions), and one circular region with a diameter covering 70% of the phantom diameter is drawn inside the phantom (signal region) as shown in Fig. 4. To calculate the SNR, the mean signal in the phantom is divided by the standard deviation of the air ROIs (*σ*_*Air*_). Since we use two air ROIs, the global standard deviation is calculated as follows: ${\sigma }_{Air}= \sqrt{({{\sigma }_{Roi1}}^{2}{{+\sigma }_{Roi2}}^{2})/2}$. The SNR test image and ROIs are shown in Fig. 4.

**Fig. 5:**
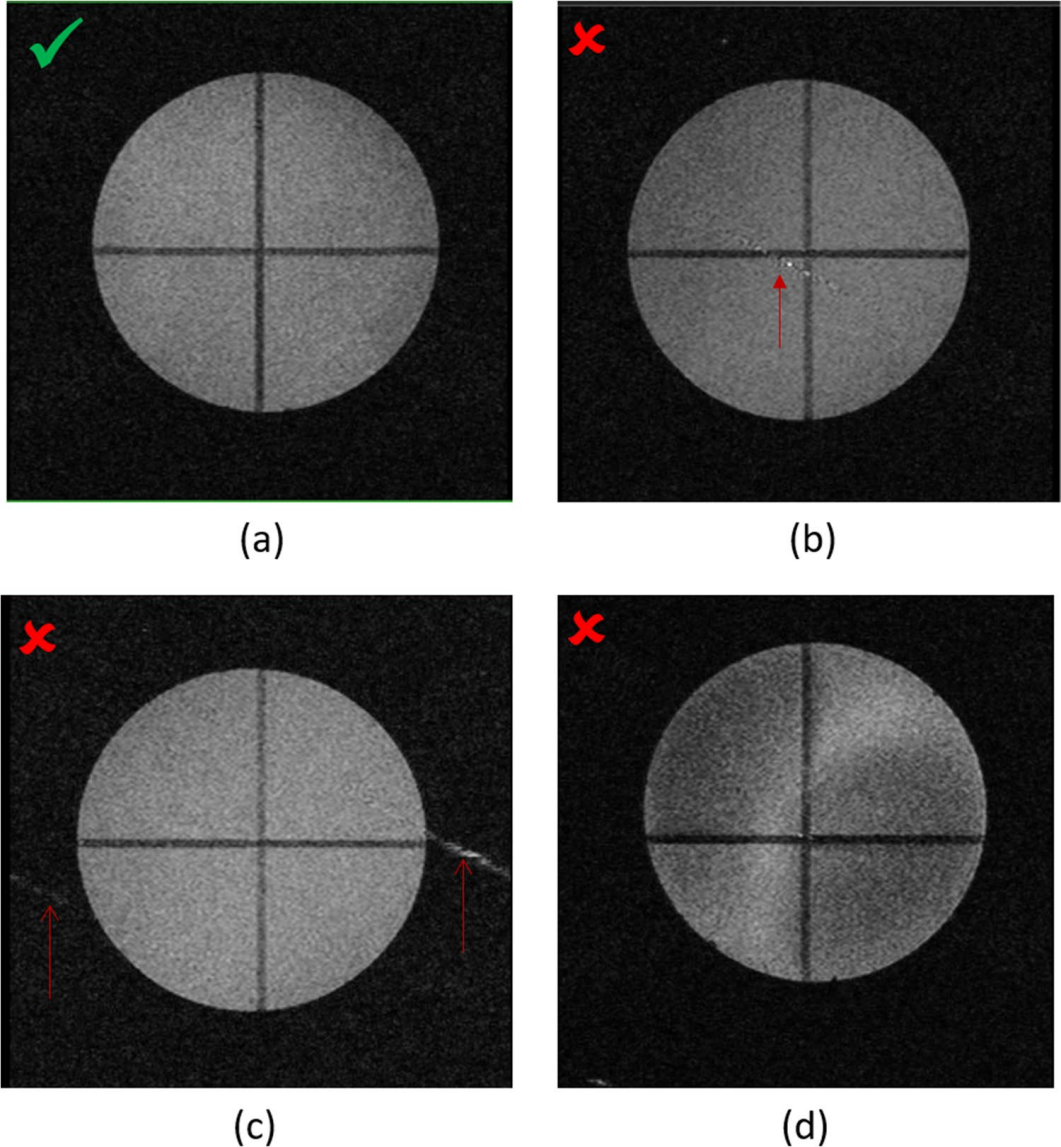
GRE transverse scouts acquired with the rat-sized homogeneous cylinder centred in the rat RF coil. (**a**) Normal image aspect. (**b**, **c**) Line artefacts (marked with a red arrow) caused by electromagnetic interferences. (**d**) Signal heterogeneities caused by damages on the RF coil to preamplifier cables. Artefact and signal issues were solved by the optimization of electromagnetic shielding and improvement in the experimental procedure

The daily procedures are summarised in Tables 1 and 3. In our system, four main types of issues were regularly encountered. The first consisted in MRI artefacts caused by electromagnetic interferences. This was easily diagnosed by the GRE scouts during the daily routine. Electromagnetic interferences can be efficiently reduced by an optimal electromagnetic shielding made of carbon or copper foils. Further actions can be useful to avoid these MR artefacts, like the removal of any unnecessary material in (or near) the FOV, a rational organisation of the imaging room limiting the electrical equipment in the vicinity, and a regular central frequency calibration. Secondly, SNR drop, signal heterogeneities and “white line” artefacts were frequently detected during the daily routine, as illustrated in Fig. 5. This was usually caused by damaged or unintentionally reversed RF coil to preamplifier cables. To limit these MRI artefacts, our RF cables are replaced every 6 months to maintain an optimal SNR. Thirdly, magnet quenches caused by a chilled water supply interruption or other equipment failures were systematically detected by the daily routine before starting the *in vivo* imaging sessions. Finally, most of the PET issues were caused by a reversible detector head issue. These were detected by the background check and most of the time solved by a PET subsystem reset.

**Table 1:** Summary of the daily checks of the QA program. To save time and highlight possible interferences, PET and MR data are acquired simultaneously

Where/when?	Check	MRI acquisition	PET acquisition	Processing	Estimated duration
When arriving in the lab	B0 check with a compass Magnet temperature Gradients chiller system Pump noise	n/a	n/a	n/a	3 min
In the magnet room	Bed heater Monitoring devices Visual inspection	n/a	n/a	n/a	3 min
Background acquisitions		Noise spectrum	Background counting rate	MR noise mean and unusual peaks Reconstruction of PET image	5–10 min
Insertion of a mouse-sized or rat-sized cylinder filled with water		Gradient echo and spin echo acquisition		Look for heterogeneity SNR, artefacts	5–10 min

### Basic Monthly Tests

#### PET High Contrast Resolution

An easy and efficient way to assess PET high contrast resolution is to use an ultra-micro hot spot phantom (UMHSP) which contains small fillable rods of increasing diameter. Even though the MR spatial resolution is smaller than the rods of the phantom, it can at least be used to check the MR spatial linearity in the axial plane. We use a UMHSP with hollow channels ranging from 0.7 to 1.2 mm filled with an activity of ^18^F in the linearity range of the system. If the linearity is not known, the activity should not be higher than the peak noise-equivalent count rate (NECR) activity [18]. On our site, the PET acquisition is carried out in list-mode for 30 min. Data are reconstructed with an ordered-subsets expectation maximisation (OSEM) algorithm, using ten iterations and 64 subsets, a voxel size of 0.28 mm × 0.28 mm × 0.28 mm, which is the smallest voxel size available on our system, and applying corrections of scatter, random, decay, normalisation, and dead time. With this reconstruction setup, the 0.7-mm rods are discernible. T2-weighted FSE slices are acquired with the following parameters: TR = 3000 ms, TE = 45 ms, matrix = 256 × 256 pixels, and FOV = 60 mm × 40 mm × 4 mm, three signal averages per acquisition.

**Fig. 6:**
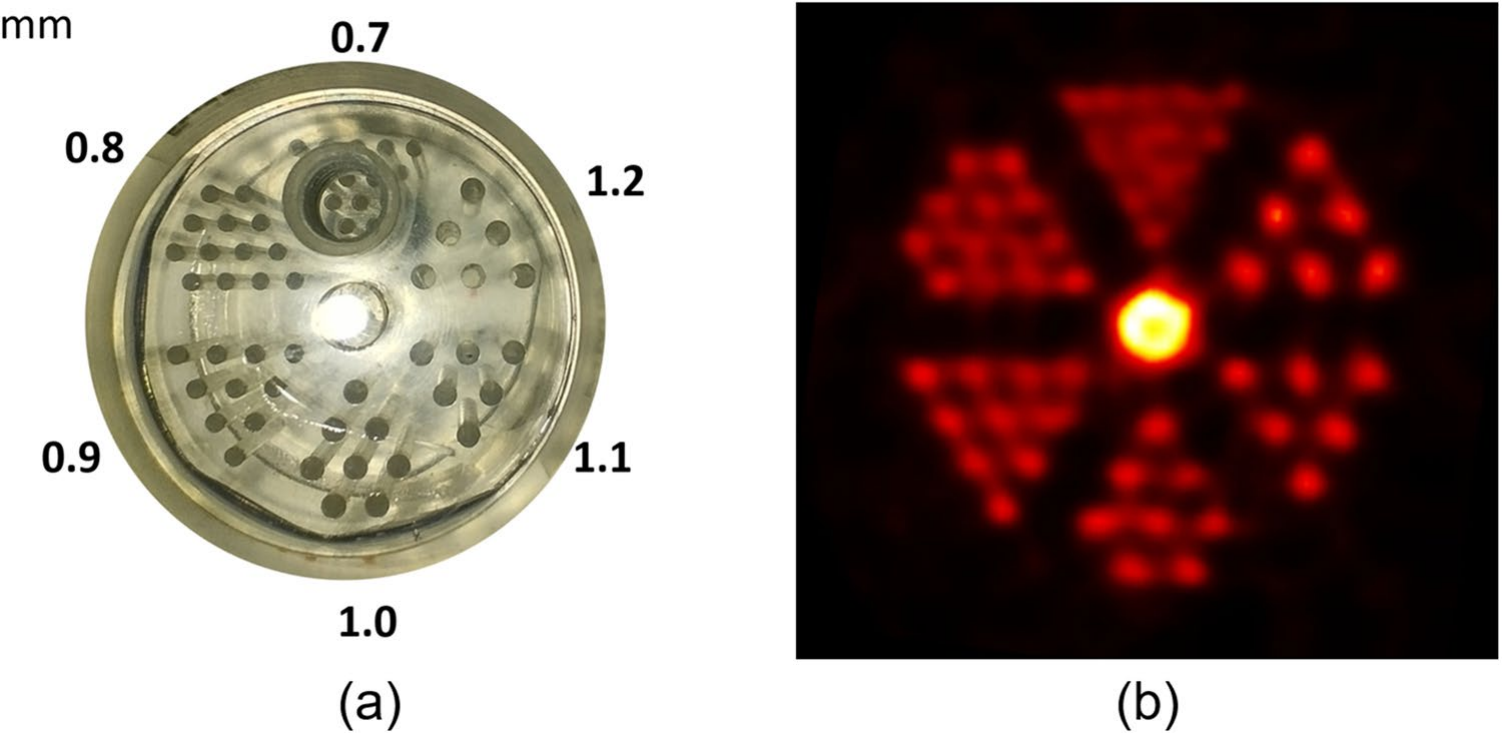
(**a**) Photography of the ultra-micro hot spot phantom used for the spatial resolution assessment displayed with the rods diameter in mm. (**b**) PET transaxial image of the ultra-micro hot spot phantom. The acquisition is 30 min long, and data are reconstructed with the OSEM algorithm using ten iterations and 64 subsets. In these conditions, the 0.8-mm rods are discernible

An overview of the produced images is shown in Fig. 6. The smallest discernible rod in each modality is reported. On MRI, one can also measure the consistency of the rod’s diameter or distance between the centre of two rods.

#### MR Geometrical Controls Tests

**Fig. 7:**
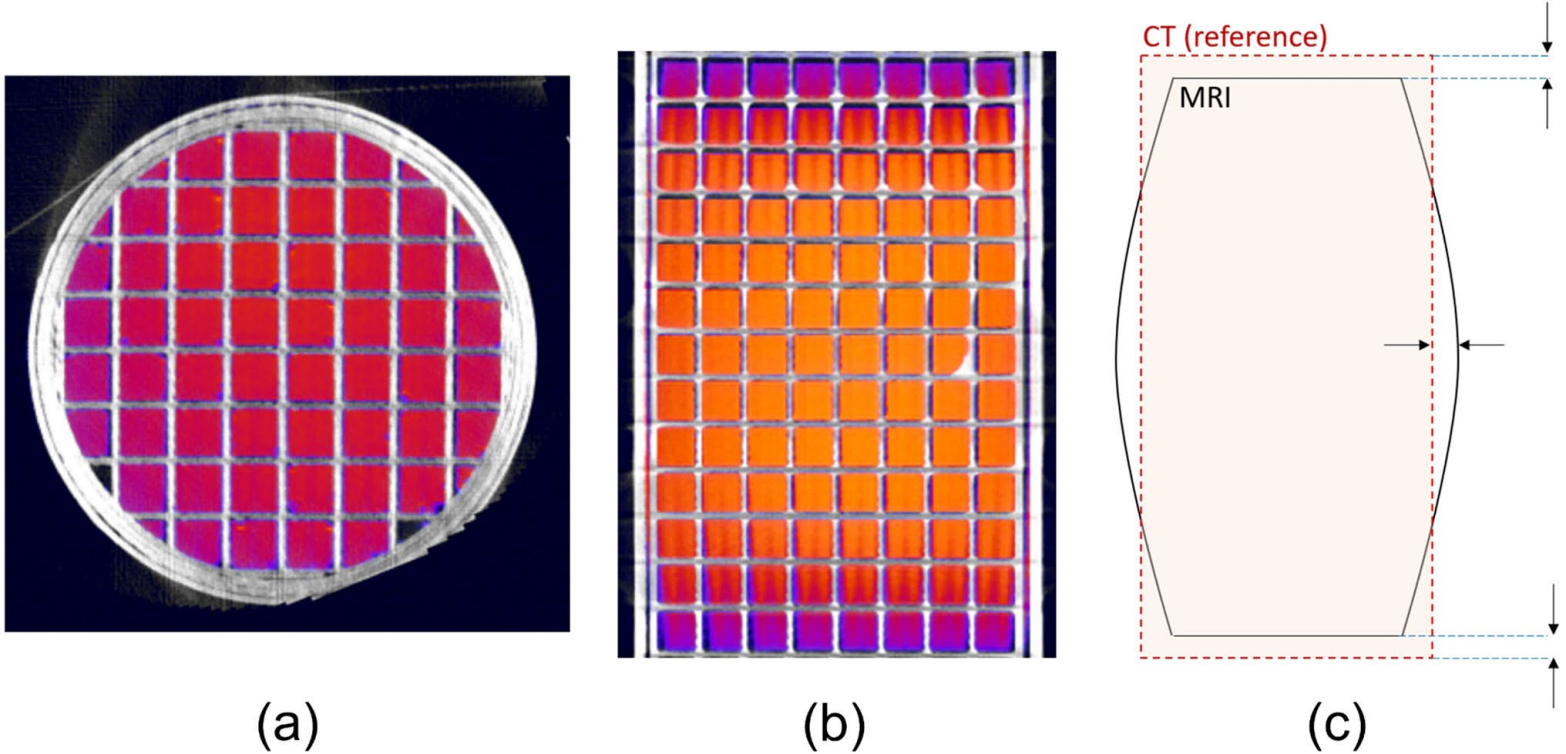
MR images (displayed in colour) of the grid phantom here registered with an X-ray micro-CT scan (displayed in grey levels). (**a**) Transaxial view. (**b**) Coronal view, where a slight distortion is visible near the MR FOV boundaries. (**c**) Schematic representation of the registration of the CT and MR images of the grid phantom in which the arrows indicate the location of the typical misalignments observed between the MR images and the CT taken as reference

B0 gradients suffer from non-linearity effects near the FOV boundaries. This results in an image deformation which is detrimental for the accurate delineation of the organs. A simple procedure to assess the spatial accuracy consists in the measurement of distances expressed as mean ± SD on several repetition by the same operator, in a 3D-printed grid phantom as the one presented in Fig. 7. One can repeat the measurement several times and over time.

Alternatively, the MR image can be overlapped with a micro-CT exam of the phantom if such scanner is available. This enhances the visibility of a possible misalignment between the two images, as shown in Fig. 7 and allows a comparison with a reference. The average percent error *E* is expressed by Eq. 1 where *L* and *l* are respectively the micro-CT (taken as reference) and micro-MR measured distances. The three space directions should be investigated.

1 $$E(\%)=100\times \frac{L-l}{L}$$

#### PET Quantification Accuracy Test

A properly calibrated PET system provides absolute quantification. This implies that the activity present in the FOV and the activity quantified in the reconstructed data should be identical within the statistical uncertainty, as long as that the acquired activity is included in the linearity range of the system. One way to follow the PET quantification accuracy is to compare on a regular basis the actual activity concentration in a phantom, obtained by a dose calibrator, expressed in MBq/g, and the corresponding activity quantified in the PET reconstructed data, expressed in MBq/mL.

A simple cylindrical phantom mimicking a mouse, with a length at least equal to the axial FOV is used for the procedure. The exact known activity of ^18^F added in the phantom should be no greater than the peak NECR activity. The acquisition and reconstruction parameters are like those applied for *in vivo* studies with ^18^F. At our site, a 30-min acquisition data is reconstructed with an OSEM algorithm using 2 iterations, 32 subsets, and an isotropic voxel size of 0.28 mm and 0.56 mm respectively for the mouse and rat phantoms. Data are corrected for scatter, random, decay, normalisation, and dead time. The activity is quantified in a volume of interest (VOI) whose size is 70% of the phantom diameter and length. The ratio between the actual and quantified activity concentrations is compared to previous results.

Beyond the quantitative analysis, the produced data can also be used to assess the image axial and transaxial homogeneities, as well as the presence of artefacts like the inter-ring distortion. If the system is designed to image other sizes of animals (rats for instance), the quantification accuracy should also be measured in a specific-sized phantom, especially if no attenuation correction (AC) is implemented on the system.

The PET quantification accuracy acquisition performed with the mouse-sized phantom has the advantage of being reusable for the energy resolution, coincidence time resolution, and crystal response map analysis as explained in the Advanced QA program section.

## Advanced QA Program

### General Principles for the Advanced QA

This section covers the monitoring of several important system characteristics that require advanced knowledge in PET and MR data acquisition and processing. Regarding the acquisition process, in addition to the previously mentioned mouse- and rat-sized cylinders, the procedure includes the use of the commercially available National Electrical Manufacturers Association (NEMA) image quality (IQ) phantom. As for the basic tests, the whole procedure is performed with PET detectors and MR coil installed inside the magnet bore.

Regarding data processing, B0 and RF fields homogeneity as well as PET NEMA IQ assessment can be completed with an in-house tool, for instance a Python program, a JAVA script executed in FiJi, or using MATLAB’s toolboxes. These tests require rather simple mathematical operations on the image matrix.

The evaluation of PET energy resolution, coincidence time resolution, and crystal response requires working with the energy, time stamps, and crystal locations of the collected coincidence events stored in a list-mode file in binary format. Vendors usually supply the adequate tools to work with these files.

The interpretation of the tests results requires an advanced knowledge of the system instrumentation and the processes involved between signal reception and final image reconstruction.

The test repetition frequency suggested thereafter should be adapted by each team depending on their use of the machine.

### Advanced Monthly Tests

The advanced monthly tests are summarised in Tables 2 and 3.

**Table 2:** Summary of the monthly program (advanced QA program)

Test	Phantom	MRI acquisition	PET acquisition/reconstruction	Processing	Estimated duration
B0 field	Mouse-sized or rat-sized cylinder	Gradient echo sequence Two echo times	n/a	ACR method	10 min
B1 field	Mouse-sized or rat-sized cylinder	Gradient echo sequence Two flip angles	n/a	Wang et al.(2006)	10 min
MR geometrical controls	Grid phantom	Fast spin echo	n/a	Measurement error on three axis	10 min
MR high contrast resolution	Ultra-micro hot spot phantom	Fast spin echo	n/a	Smallest discernible rods	10 min
PET quantification accuracy	Mouse-sized and/or rat-sized cylinder	n/a	30 min acquisition in list-mode OSEM 2 iterations, 32 subsets	Scanner-dose calibrator cross calibration	1 h per phantom
PET spatial resolution	Ultra-micro hot spot phantom	n/a	30 min acquisition in list-mode OSEM 2 iterations, 32 subsets	Smallest discernible rods	1 h
PET energy resolution	Mouse-sized or rat-sized cylinder	n/a	Use quantification accuracy data	In-house tool	15 min
PET coincidence time resolution		n/a			
PET crystal response		n/a			
PET image quality	NEMA IQ	n/a	20 min List-mode, OSEM 2 iterations, 32 subsets	NEMA NU4 2008 processing	1 h

**Table 3:** Overview of the acquisition procedure of the QA protocol. The mouse- and rat-sized cylinders are respectively 2 cm and 4 cm in diameter, and 10 cm in length. The mouse and rat body coils inner diameter are respectively 3 cm and 6.7 cm. The 3D printed MR geometrical controls phantom contains an alignment of 5-mm squares filled with water. O’Callaghan et al. [19] provided the 3D prints plan of a similar phantom

Modality	Frequency	Test	Phantoms		RF coil	Simultaneous scan?
MRI	Daily	SNR (SE image) Image homogeneity and artefacts (GRE scout) System noise (NMR spectrum)	Mouse-sized homogeneous cylinder	Custom manufacturing	Mouse body coil	PET background acquisition
	Monthly	B0 homogeneity (GRE images, two TE) B1 homogeneity (GRE images, two flip angles)	Rat-sized homogeneous cylinder	Custom manufacturing	Rat body coil	PET quantification accuracy (rat)
		MR high resolution	UMHSP	Commercially available	Mouse body coil	PET spatial resolution
		MR geometrical controls	Grid phantom	3D printed	Rat body coil	n/a
PET	Daily	Background acquisition	Mouse-sized cylinder	Custom manufacturing	Mouse coil	MR daily tests
	Monthly	Quantification accuracy (mouse)	Mouse-sized homogeneous cylinder	Custom manufacturing	Mouse body coil	B0 and B1 homogeneity
		Quantification accuracy (rat)	Rat-sized homogeneous cylinder	Custom manufacturing	Rat body coil	B0 and B1 homogeneity
		Spatial resolution	UMHSP	Commercially available	Mouse body coil	MR high resolution
		Energy resolution	Mouse- or rat-sized cylinder	Custom manufacturing	Mouse or rat body coil	B0 and B1 homogeneity
		Coincidence time resolution	Mouse- or rat-sized cylinder	Custom manufacturing	Mouse or rat body coil	B0 and B1 homogeneity
		Crystal response	Mouse- or rat-sized cylinder	Custom manufacturing	Mouse or rat body coil	B0 and B1 homogeneity
		Image quality	NEMA IQ phantom	Commercially available	Rat body coil	n/a
	Annually	Registration	In-house object	(syringes)	Mouse or rat body coil	MR registration test images
		NEMA spatial resolution	^22^Na Point source	Commercially available	Mouse body coil	n/a
		NEMA count rates (mouse)	NEMA count rates (mouse)	Commercially available	Mouse body coil	n/a
		NEMA count rates (rat)	NEMA count rates (rat)	Commercially available	Rat body coil	n/a
		NEMA sensitivity	^22^Na Point source	Commercially available	Mouse body coil	n/a

#### MR Principal Magnetic Field Homogeneity Test

**Fig. 8:**
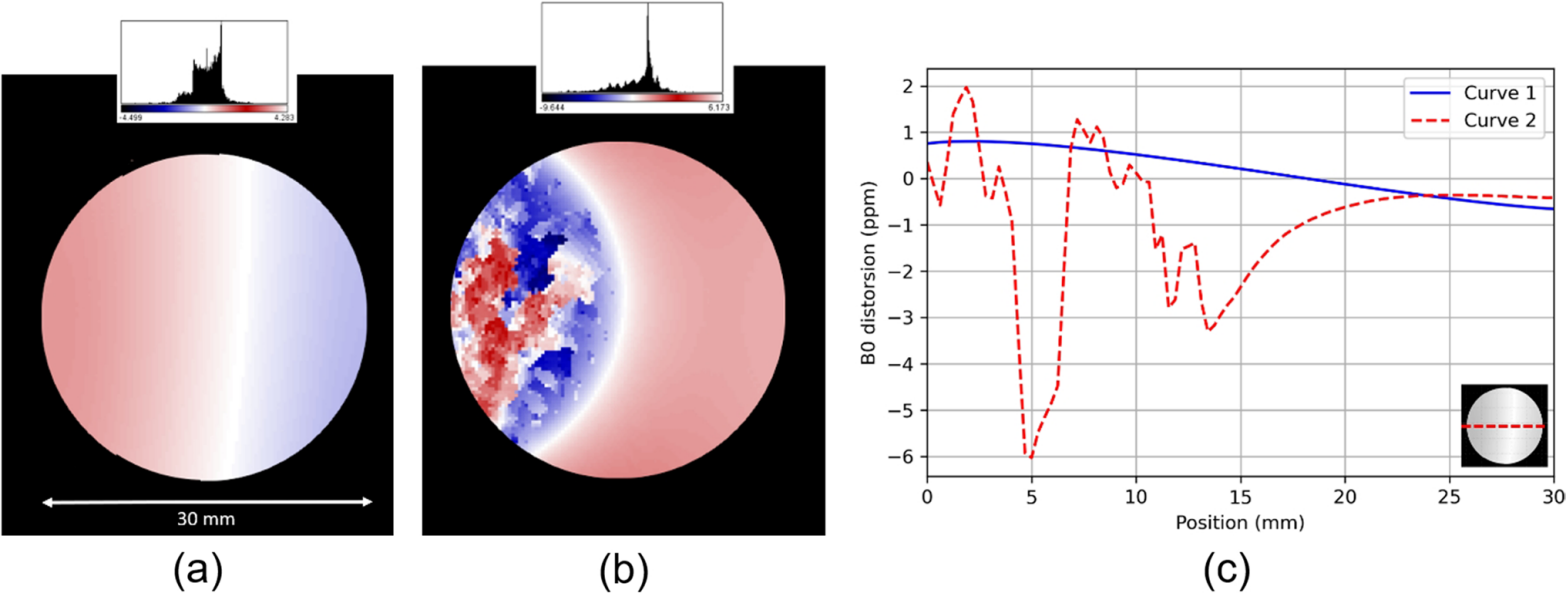
(**a**) An example of transverse B0 distortion maps (ppm) obtained in our 7-Tesla magnet. (**b**) The effect of a non-MR compatible catheter voluntarily introduced in the FOV. The acquisition procedure remains unchanged. (**c**) A linear horizontal profile traced on the (**a**) and (**b**) maps. Curve number 2 shows the B0 distortion induced by the presence of the catheter compared to curve number 1 obtained after removing it

Some applications like functional MRI and NMR spectroscopy require a highly homogeneous B0. The procedure and underlying theory are described in detail in the ACR guide [17]. The suggested measurement workflow can be summarised as follows. The rat-sized water cylinder is installed in a large quadrature coil. A dual echo time GRE image is acquired with the following parameters: TR = 1000 ms, TE = 3.14 ms (TE1) and 7.62 ms (TE2), image matrix = 128 × 128 pixels, and FOV = 60 mm × 40 mm × 4 mm, flip angle = 30°, three signal averages per acquisition. Phase images are calculated from the raw MR data. The phase is then unwrapped using a 2D phase unwrapping method [20]. Finally, a subtraction of the two-phase images is performed to create a phase-difference map, where the values are proportional to the spin precession difference. Once converted in field variation map using the Larmor relation, the phase-difference map depicts the local B0 heterogeneity, expressed in part per million (ppm) of B0. The mean ± SD of the B0 variation in a 3-cm diameter ROI and the maximum and minimum values within a 6-cm long profile following the phase variation direction are reported. The procedure is repeated in the axial and coronal planes. The ppm values are ideally as low as possible, or at least close to the supplier’s specifications. Typically, a distortion of less than 1 or 2 ppm in a 3-cm diameter sphere should be observed in most magnets. An example is proposed in Fig. 8.

#### Radiofrequency Coil Test

In addition to the measurement of B0 homogeneity, it is also important to control the homogeneity of the radiofrequency field, also called B1. The SNR measurement presented in the basic QA program section is a way to control the absorbed power in the FOV. The flip angle homogeneity is another important characteristic. Indeed, in a longitudinal relaxation time or relaxation rate measurement (T1 or R1 mapping) made with a fast GRE sequence like fast-low angle shot (FLASH), the applied flip angle accuracy is a critical point [21]. We apply the method proposed by Wang et al*.* to investigate the flip angle homogeneity [22].

A phantom adapted to the RF coil used for the *in vivo* experimentation is installed. Two GRE images are acquired using a flip angle *α* = 45° in acquisition 1 and a flip angle *α* = 90° (twice the first flip angle) in acquisition 2. The other acquisition parameters are TR = 1000 ms, TE = 5 ms, matrix = 128 × 128 pixels, and FOV = 60 mm × 40 mm × 4 mm, three signal averages per acquisition. The signal *S*1 and *S*2 respectively measured during acquisition 1 and acquisition 2 are combined with Eq. 2 to produce a flip angle map expressed in degrees. This map reflects the B1 homogeneity. Signal mean ± SD over the ROI is measured on the flip angle map in a 3-cm diameter central circular region. The flip angle variation along a linear profile is also reported.

2 $${\alpha }_{\mathrm{exp}}={\mathrm{cos}}^{-1}\left|\frac{{s}_{2}}{2{s}_{1}}\right|$$

Ideally, flip angle values should be close to *α* = 45° at any point of the map. Unexpected heterogeneous areas of the flip angle map should be addressed.

#### PET Image Quality Test

The NEMA IQ measurement [18] is suitable for a quick and easy monitoring of the main PET image quality indexes: the non-uniformity coefficient measured in a central uniform region, the recovery coefficients (RC) calculated in radioactive rods, and the two spill over ratios (SOR) measured in air- and water-filled cold regions.

The phantom is filled with an accurately known activity of ^18^F, and a 20-min PET list-mode acquisition is started once the activity reaches 3.7 MBq. For successful acquisitions, the ^18^F concentration must be homogeneous, and any air bubbles avoided. At our site, images are reconstructed with the parameters used *in vivo*, which uses an OSEM algorithm with two iterations, 32 subsets, a voxel size of 0.42 mm × 0.42 mm × 0.42 mm, and corrections of scatter, random, decay, normalisation, and dead time.

**Fig. 9:**
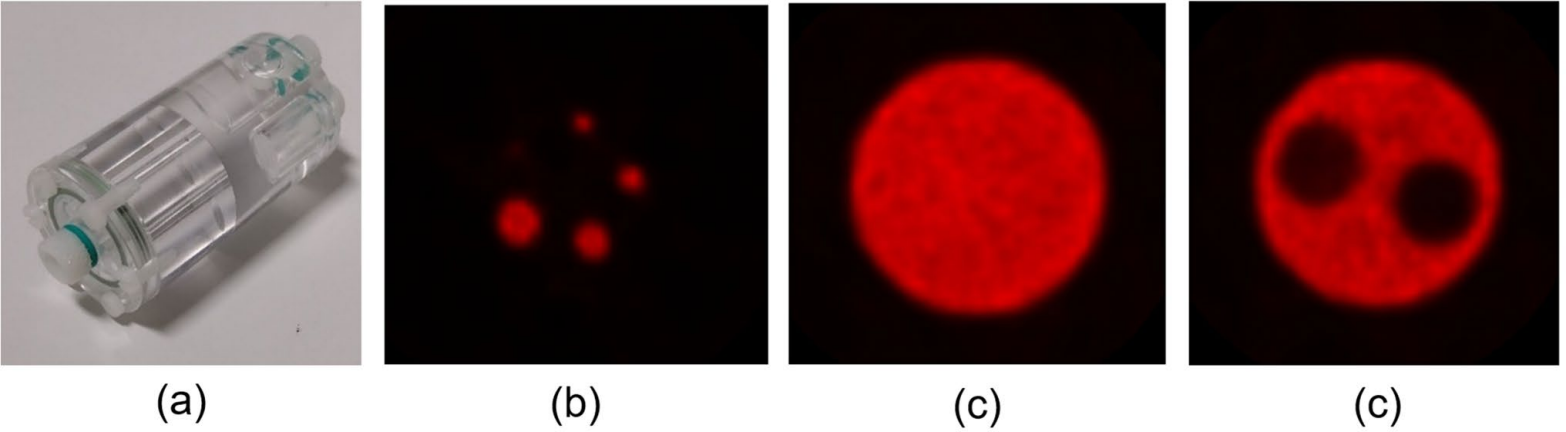
(**a**) A picture of the NEMA IQ phantom. (**b**) The recovery coefficients insert of the phantom. (**c**) The homogeneity insert of the phantom. (**d**) The spill-over ratio region

The images are analysed with an in-house software. RC, non-uniformity, and SORs are calculated from data. The complete analysis workflow is detailed in the NEMA standard [18]. The non-uniformity coefficient reports the SNR of reconstructed PET data, whereas RCs and SORs provide information respectively on the system point spread function and the raw data corrections. Images of the NEMA IQ phantom are given in Fig. 9. To interpret these figures, one should refer to the system acceptance results or to the supplier’s specifications, if measurements were made complying with the NEMA standard.

#### PET Energy and Coincidence Time Resolutions (CTR) Tests

The energy resolution is an essential characteristic of detectors relying on energy spectroscopy. It reports the ability of the detector to separate the useful energy fraction from the noise contribution. CTR characterises the detection time difference between the two photons of a coincidence. PET energy and CTR are assessed from the list-mode data acquired on the mouse-sized phantom during the quantification accuracy test, for which the detectors are uniformly irradiated. Calculations are usually performed with the software provided by the vendor, or alternatively with an in-house software.

The energy resolution, expressed in percent, is defined as the photopeak full width at half maximum (FWHM) in keV divided by the photopeak position in the energy histogram of a detection head. The system energy resolution is defined as the mean ± SD of the energy resolutions of the detection heads.

A coincidence time histogram is generated on direct opposite pairs of detectors from the event time stamps written in the list-mode data. The coincidence time resolution, expressed in nanosecond (ns), is defined as the coincidence time histogram FWHM.

#### PET Detectors Response Test

**Fig. 10:**
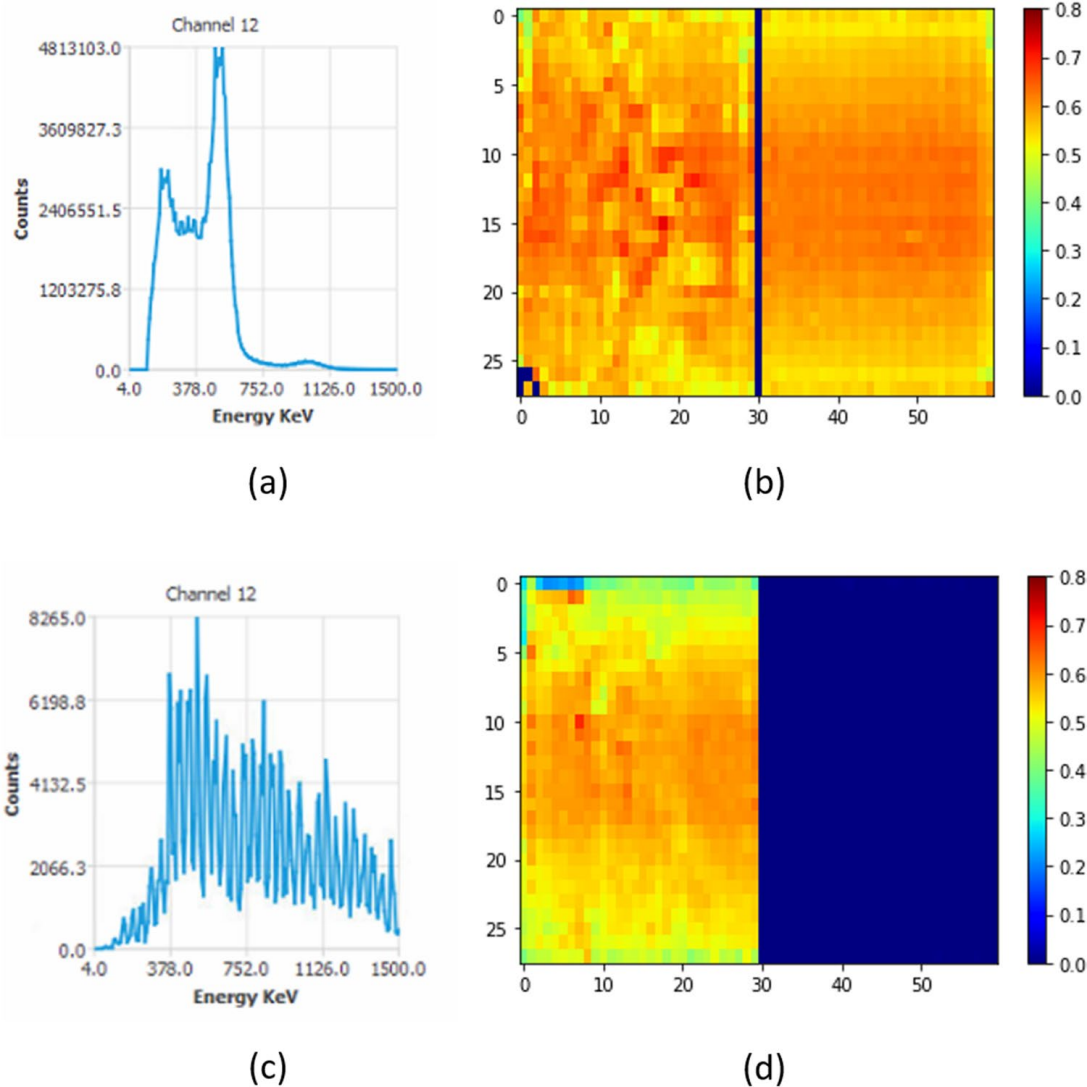
(**a**) An example of energy spectrum (counts per keV) acquired with our system. The measured energy resolution is about 20% with a 1-cm deep LYSO-CE crystal. (**b**) A dual-layer crystal response map normalised to the detector maximum count, showing acceptable heterogeneous areas. (**c**), (**d**) Non-compliant results obtained on the same detector due to an electric supply failure of an SiPM tile. A system reset fixed the issue

Crystals are fragile elements which can deteriorate under the influence of external perturbations like moisture or vibrations. A deterioration of the crystal or the associated SiPM elements and electronics can lead to artefacts and inaccurate quantitative data. Hence, the integrity of every detector head must be monitored. To do so, a detector response map to a uniform irradiation is generated from the list mode data of the PET quantification accuracy acquisition on the mouse-size phantom. This map can highlight a failure in the detection chain. Some examples of results are provided in Fig. 10.

### Advanced Annual Measurements

**Fig. 11:**
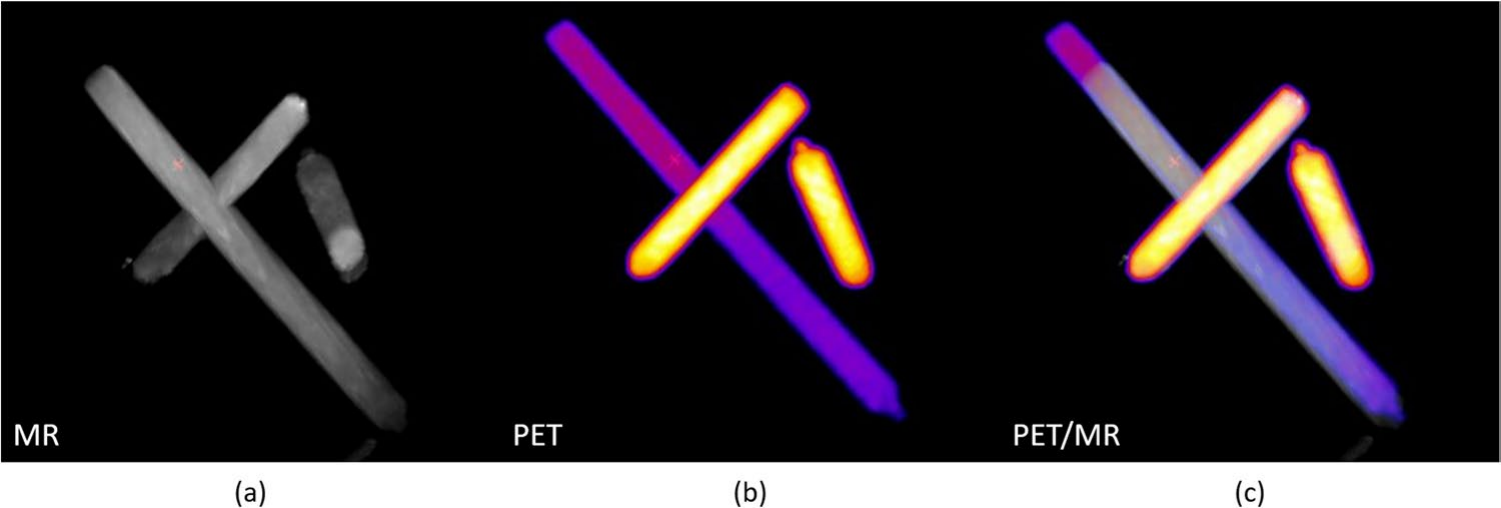
Overview of the PET/MR image registration accuracy test object used in our facility. The object is made of three 1-mL syringes filled with 10 MBq of ^18^F. Simultaneously, acquired PET and MR datasets are exported in DICOM before being displayed *in Vivo*Quant 2.0 (Invicro, Needham, MA, USA). MR images were resampled to the PET isotropic voxel size of 0.56 mm. (**a**) The maximum intensity projection (MIP) of the MR image (axial FOV of 8 cm). (**b**) The MIP of the PET image (axial FOV of 10 cm). (**c**) The MIP of the PET/MR registration

In an integrated system, the PET/MR registration should be straightforward provided that the images localization is properly encoded in the DICOM headers. This is usually checked during the system acceptance procedure. It is also important to verify the absence of any drift in the registration of PET and MR datasets. This can be easily performed using the MR geometrical test phantom filled with radioactivity or another test object giving signal in the two modalities. We use an asymmetrical in-house object made of three 1-mL syringes filled with a ^18^F solution. An overview of the resulting images is provided in Fig. 11. We would suggest performing this test once a year, of after every major hardware or software modification on the system [23].

The sensitivity, counting rates, image quality, and spatial resolution measurements of the NEMA-NU4-2008 standard [18] can be used for a long-term instrumental follow-up with an annual periodicity. Sensitivity and counting rates indexes report the event counting efficiency of the system. These characteristics mainly depend on the system detection solid angle, crystal material and depth, and SiPM technology. The NECR can be seen as an index of the PET raw data SNR as a function of counting rates.

## Discussion and Conclusion

The present paper has suggested a set of tests designed to help running reliable experiments. In the last 5 years, these QA procedures prevented most of the malfunctions encountered in the biological experiments performed in our facility. Usually, the detected failures are quickly resolved by very simple measures such as resetting computers and electronics or checking cables and their connections. For that reason, the procedures should be short enough to avoid delaying the biological experiments. Thus, comprehensive procedures like those described in the existing NEMA standard for preclinical PET alone system are not adapted to frequent (daily) and quick tests. Although not included in standard procedures, the simple acquisition of the background signal proved to be very sensitive to usual malfunctions for both MR and PET.

Regarding tests with lower periodicity, the stability of the scanner performance in time has to be considered. In our experience, PET characteristics appear to be quite stable in time and most PET issues can be easily solved. The same is not true for MR systems, particularly for those that are not surrounded by a Faraday cage. Having used a 7-T superconducting magnet for several years, we observed that MR image quality is likely to drift with time. Finding the origin of an image artefact or an unwanted NMR frequency peak is sometimes not trivial. Therefore, MRI tests should be performed with a periodicity of at least 1 month. We would even recommend a weekly repeated measurement of the magnetic fields homogeneity and SNR for teams dealing with quantitative or functional MRI. No recommendations are available for preclinical performance assessment in MR even as a standalone modality. The MR tests suggested in the present paper are derived from the recommendation of the American College of Radiology for clinical magnetic resonance assessment and are close to those used for performance assessment in a previous work [5]. Moreover, SNR, B0, and B1 homogeneity tests do not require sophisticated phantoms neither radioactivity manipulation, making these tests easy to perform.

PET tests described in the NEMA procedures for preclinical PET are somewhat cumbersome, and alternative procedures were developed in the present study. For example, regarding spatial resolution assessment, the NEMA test was replaced by a hot-spot phantom analysis, easy to set up and compatible with iterative reconstructions used *in vivo* as opposed to the filtered back projection recommended in the NEMA procedures. SiPM failure described in Fig. 10, may have been unnoticed in a living animal. In these situations, whereas the image looks qualitatively acceptable, the quantified activity may be underestimated. In addition, the quantification accuracy follow-up is required to ensure the quantitative aspects of data. However, it has to be acknowledged that NEMA tests remain the unsurpassed basis for acceptance tests, intercomparison between different scanners and system long-term follow-up. We recommend to perform these tests only at the acceptance of the scanner and thereafter once a year.

One of the requirements for quality assessment is the availability of phantoms suitable for the transaxial FOV which is limited by the presence of the PET detectors. Very simple objects such as cylindrical phantoms or commercially available hotspot phantoms allow the most common tests to be performed. For more sophisticated approaches, processes such as stereolithography supported by 3D printers allow the creation of small-dimension phantoms at reasonable costs [20]. These phantoms are very useful to fit to specific tests. In the near future, this could be a solution to set up a pattern phantom dedicated to high- and low-contrast resolutions in MRI or to design phantoms specifically adapted to test the accuracy of image registration.

From registered PET and MR datasets, an accurate MR-based attenuation correction is mandatory to ensure the quantitative aspect of PET data. The procedures for attenuation correction vary according to the animal (rat or mouse), the organ or tissue concerned (brain, thorax, abdomen, limbs), and the MR materials present in the PET field of view. To our knowledge, there is currently no standard nor reference methodology to perform such correction in the preclinical domain. Therefore, quality assessment is not straightforward and will also be highly dependent of the animal size and organ considered. Although of critical importance, this aspect of quality assessment is beyond the scope of this article focused on QA of data acquisition.

Mutual interference between subsystems is also a topic of interest when using integrated PET/MR systems [15]. It is recommended that QA tests be performed in the presence of equipment from both modalities and, ideally, with both modalities running. Several consequences of simultaneous acquisitions on PET and MR data can be considered. Regarding the effect of MRI on PET, sequences that make extensive use of magnetic field gradient coils may result in quantitative data drift if the ability of the system to correct for temperature variations is exceeded. Moreover, any material installed between the source and the PET detector (RF coils, animal cradle, wires) may increase the scattering and attenuation of photons, thus affecting the PET performance. As a consequence, one should at least perform a PET quantification accuracy test in both standalone and simultaneous modes when implementing new MR sequences or installing new devices in the FOV.

Regarding the effect of PET on MRI, radiated or conducted electromagnetic interference causing SNR modulation or artefacts should be investigated with homogeneous phantom acquisitions. For instance, we [5] and others [24, 25] noticed a moderate SNR decrease within our MR images when simultaneously scanning at high activity in the FOV. In case of other radiated or conducted electromagnetic interferences affecting the MR system and causing artefacts, the magnet’s direct environment should be checked, especially regarding the presence of electronic devices.

Recommendations do exist for standalone clinical PET and MR [17, 23], and several initiatives [24–26] are paving the way to the standardisation of clinical hybrid systems. In the preclinical domain, to our knowledge, the available recommendations deal with standalone PET systems [23]. Preclinical PET/MR imaging deserves such an approach. Indeed, as stated by McDougald et al. [27], the standardisation of QA is a further step towards the reliability, reproducibility, validity, and translatability of acquired data. The present paper aims at offering a contribution towards preclinical PET/MR QA with a strong focus on ease of implementation in the workflow of a preclinical facility.

The QA procedure detailed in this article allows the detection of the most frequent issues in both modalities and ensures the quantitative aspect of PET and MR data. The procedure intends to provide a panel of efficient methods to anticipate the most probable performance drifts. It has to be amended by teams depending on the specific requirements of their research context.

Finally, a significant gap exists between a simple phantom acquisition and an *in vivo* experimentation where anatomical complexity and cardiorespiratory movements must be dealt with. Therefore, the *in vivo* data quality and possible artefacts should be continuously inspected all along the system lifetime.

## References

[1] Delso G, Ziegler S (2009). PET/MRI system design. Eur J Nucl Med Mol Imaging.

[2] Christen T, Bouzat P, Pannetier N (2014). Tissue Oxygen Saturation Mapping with Magnetic Resonance Imaging. J Cereb Blood Flow Metab.

[3] Jahng G-H, Li K-L, Ostergaard L (2014). Perfusion Magnetic Resonance Imaging: A Comprehensive Update on Principles and Techniques. Korean J Radiol.

[4] Boellaard R (2009). Standards for PET Image Acquisition and Quantitative Data Analysis. J Nucl Med.

[5] Courteau A, McGrath J, Walker PM (2021). Performance Evaluation and Compatibility Studies of a Compact Preclinical Scanner for Simultaneous PET/MR Imaging at 7 Tesla. IEEE Trans Med Imaging.

[6] Ko GB, Yoon HS, Kim KY (2016). Simultaneous Multiparametric PET/MRI with Silicon Photomultiplier PET and Ultra-High-Field MRI for Small-Animal Imaging. J Nucl Med.

[7] Lecomte R (2009). Novel detector technology for clinical PET. Eur J Nucl Med Mol Imaging.

[8] Peng H, Levin CS (2010). Recent Developments in PET Instrumentation. Curr Pharm Biotechnol.

[9] Stortz G, Walker MD, Thompson CJ (2013). Characterization of a New MR Compatible Small Animal PET Scanner Using Monte-Carlo Simulations. IEEE Trans Nucl Sci.

[10] Ko GB, Kim KY, Yoon HS (2015). Evaluation of a silicon photomultiplier PET insert for simultaneous PET and MR imaging: Silicon photomultiplier PET insert for simultaneous PET/MRI. Med Phys.

[11] Wehner J, Weissler B, Dueppenbecker PM (2015). MR-compatibility assessment of the first preclinical PET-MRI insert equipped with digital silicon photomultipliers. Phys Med Biol.

[12] Omidvari N, Cabello J, Topping G (2017). PET performance evaluation of MADPET4: a small animal PET insert for a 7 T MRI scanner. Phys Med Biol.

[13] Gsell W, Molinos C, Correcher C (2020). Characterization of a preclinical PET insert in a 7 tesla MRI scanner: beyond NEMA testing. Phys Med Biol.

[14] Emvalomenos G, Trajanovska S, Pham BTT (2021). Performance evaluation of a PET insert for preclinical MRI in stand-alone PET and simultaneous PET–MRI modes. EJNMMI Phys.

[15] Truhn D, Kiessling F, Schulz V (2011). Optimized RF shielding techniques for simultaneous PET/MR: Optimized RF shielding techniques for simultaneous PET/MR. Med Phys.

[16] Grage H, Akke M (2003). A statistical analysis of NMR spectrometer noise. J Magn Reson.

[17] Price R, Allison J, Clarke G et al (2015) Magnetic Resonance Imaging Quality Control Manual of the American College of Radiology Committee on Quality Assurance in Magnetic Resonance Imaging

[18] National Electrical Manufacturers Association (2008) Performance measurements of small animal positron emission tomographs. In: NEMA Standards Publication No. NU 4-2008, National Electrical Manufacturers Association, Rosslyn, VA

[19] O’Callaghan J, Wells J, Richardson S (2014). Is Your System Calibrated? MRI Gradient System Calibration for Pre-Clinical. High-Resolution Imaging PLoS ONE.

[20] Herráez MA, Burton DR, Lalor MJ (2002). Fast two-dimensional phase-unwrapping algorithm based on sorting by reliability following a noncontinuous path. Appl Opt.

[21] Jenkins C, Papadopoulos I, Shermer SM (2019) Comparison of R1 Mapping Protocols: What are we measuring? arXiv:1909.12984

[22] Wang J, Mao W, Qiu M (2006). Factors influencing flip angle mapping in MRI: RF pulse shape, slice-select gradients, off-resonance excitation, andB0 inhomogeneities. Magn Reson Med.

[23] Busemann Sokole E, Płachcínska A, Britten A (2010). Routine quality control recommendations for nuclear medicine instrumentation. Eur J Nucl Med Mol Imaging.

[24] Matheoud R (2022) Quality controls in PET/CT and PET/MRI, EFOMP’S Guidelines

[25] Koole M, Armstrong I, Krizsan AK et al (2022) EANM guidelines for PET-CT and PET-MR routine quality control. EANM Physics committee, Zeitschrift für Medizinische Physik 10.1016/j.zemedi.2022.08.003 PMC10068535 36167600

[26] Valladares A, Ahangari S, Beyer T (2019). Clinically Valuable Quality Control for PET/MRI Systems: Consensus Recommendation From the HYBRID Consortium. Front Phys.

[27] McDougald W, Vanhove C, Lehnert A (2020). Standardization of Preclinical PET/CT Imaging to Improve Quantitative Accuracy, Precision, and Reproducibility: A Multicenter Study. J Nucl Med.

